# Right ventricular pressure-volume loop analysis in congenital heart disease

**DOI:** 10.1016/j.ijcchd.2025.100625

**Published:** 2025-10-02

**Authors:** Renée S. Joosen, Michael G. Dickinson, Marielle C. van de Veerdonk, Rahi S. Alipour Symakani, Daphne Merkus, Michiel Voskuil, Gregor J. Krings, Johannes M.P.J. Breur

**Affiliations:** a Department of Pediatric Cardiology, University Medical Center Utrecht, Utrecht, Netherlands; b Department of Cardiology, University Medical Center Utrecht, Utrecht, Netherlands; c Department of Cardiology, Amsterdam University Medical Centers, Amsterdam Cardiovascular Sciences, University of Amsterdam, Amsterdam, Netherlands; d Department of Pediatrics, Division of Pediatric Cardiology, Erasmus Medical Center, Sophia Children's Hospital, Rotterdam, Netherlands; e Department of Cardiology, Division of Experimental Cardiology, Erasmus Medical Center, Rotterdam, Netherlands; f Department of Cardiothoracic Surgery, Erasmus Medical Center, Rotterdam, Netherlands; g Institute for Surgical Research, Walter Brendel Center of Experimental Medicine (WBex), University Clinic Munich, Munich, Germany; h German Center for Cardiovascular Research (DZHK), Partner Site Munich, Munich Heart Alliance (MHA), Munich, Germany

**Keywords:** Congenital heart defects, Right ventricle, Heart catheterization, Hemodynamics

## Abstract

**Background:**

Right ventricular (RV) function is an independent predictor of prognosis in patients with congenital heart disease (CHD). Early detection is therefore crucial but current methods to assess RV function are highly load-dependent. RV pressure-volume (PV) loop analysis is an invasive technique which is load-independent and considered the gold standard for evaluating RV adaptation in relation to its load. However, RV PV loop analysis can be time consuming and prone to inter-operator variability.

**Methods:**

This article provides a comprehensive guide on performing RV PV loop analysis using the multi-beat method in pediatric and adult patients with complex CHD.

**Results:**

RV PV loop analysis using the multi-beat method can be safely and reliably applied in children and adults with complex CHD.

**Conclusion:**

RV PV loop analysis offers a valuable tool for advanced insight into RV adaptation and a deeper understanding of cardiac physiology in complex CHD patients with RV pressure and/or volume overload. By enhancing our understanding of RV adaptation, this approach holds great potential for improving patient management and optimizing treatment strategies.

## Highlights

• RV PV loop analysis via multi-beat method is safe and reliable in complex CHD patients.
• RV PV loops give advanced insight into RV adaptation to pressure/volume overload in CHD patients.
• Improved insight in RV adaptation may enhance patient care and optimize treatment in CHD.

## 1 Background

**Fig. 1:**
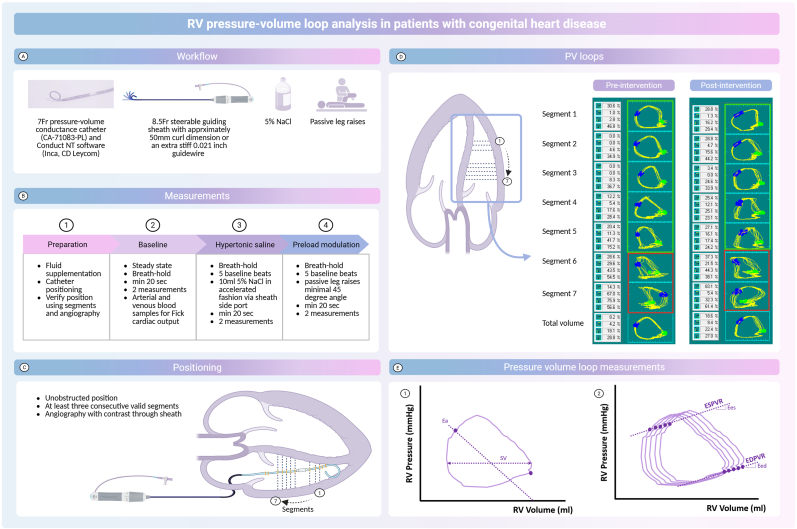
**RV pressure-volume loops in congenital heart disease patients** (A) Workflow of RV PV loop analysis, (B) Pre and post-interventional RV PV loop analysis measurements, (C) positioning of the conductance catheter,(D) pre- and post-intervention PV loops from a patient with transposition of the great arteries after arterial switch operation with RV pressure overload due to pulmonary artery stenosis and (E) a schematic representation of pressure volume loop measurements. Red = bad segments (excluded), green = good segments. Created in BioRender. Joosen, R. (2025) https://BioRender.com/q70q580. Ea = arterial elastance; Eed = end-diastolic elastance; Ees = end-systolic elastance; PV = pressure-volume, RV = right ventricle; SV = stroke volume.

Right ventricular (RV) function is an independent predictor of prognosis in patients with congenital heart disease (CHD) [1]. Chronic RV pressure or volume overload can cause RV maladaptation, progressing to dysfunction, failure, reduced quality of life and mortality [2]. Early detection is crucial, but current methods to assess RV function are load-dependent. RV pressure-volume (PV) loop analysis is an invasive technique which is load-independent and considered the gold standard for evaluating RV adaptation in relation to its load [3]. However, performing RV PV loop analyses using the multi-beat method can be time consuming and prone to inter-operator variability. This article provides a comprehensive guide on performing RV PV loop analysis using the multi-beat method in pediatric and adult patients with complex CHD, including conditions like transposition of the great arteries (TGA) post-arterial switch operation (ASO) and tetralogy of Fallot (ToF) (Fig. 1). These patients frequently experience RV pressure and/or volume overload due to residual lesions following surgical or percutaneous interventions.

## 2 Methods and results

### 2.1 Workflow

All patients undergo right heart catheterization (RHC) under general anesthesia, with central venous and arterial access, electrocardiography, and pulse oximetry monitoring, although it can also be performed under local anesthesia when appropriate. RV PV loop analysis is conducted using a 7Fr pressure-volume conductance pigtail catheter, with a solid-state pressure sensor and twelve 8 mm-spaced electrodes (catheter type: CA-71083-PL, CD Leycom, Hengelo, the Netherlands) along with Conduct NT software (version 3.18.1, Inca, CD Leycom). An extra stiff 0.021 inch guidewire or a 8.5Fr steerable guiding sheath with approximately 50 mm curl dimension are required for catheter positioning. Additionally, two 10 ml doses of hypertonic saline (5 % NaCl) are required for volume calibration and an independent measurement of stroke volume. All measurements are performed pre and post intervention.

### 2.2 Preparation and positioning of the conductance catheter

General anesthesia and preoperative fasting can lead to hypovolemia. Patients should be encouraged to continue drinking clear fluids preoperatively (e.g., water, apple juice) to prevent hypovolemia. In addition, fluid supplementation with saline (15 ml per kg for children, 300–500 ml for adults) or a contrast agent administered during 3D rotational angiography (approximately 100 ml/s, with ¼ to ⅓ contrast and the remainder saline) is recommended [4]. The conductance catheter is soaked in wet gauze during connection to the Inca system. Subsequently, it is inserted under fluoroscopic guidance via femoral access (using a 8.5Fr steerable guiding sheath to position the pigtail conductance catheter in the RV apex since the catheter is too stiff to cross the tricuspid valve with a 0.021 guidewire) or jugular access (short 7Fr sheath, using the extra stiff 0.021 inch guidewire) and positioned at the RV apex, ensuring it remains unobstructed and free from trabeculations or the moderator band. Jugular access, advantageous in case of inferior vena cava occlusion, facilitates easier catheter positioning by placing the extra-stiff wire in the RVOT and allowing the conductance catheter to “fall” into the RV apex.

### 2.3 Mechanism of action

An electrical current between distal and proximal electrodes creates an electronic field. Due to blood's conductivity, voltage drops across each electrode pair, with the magnitude of the voltage difference being inversely proportional to the RV's cross-sectional area at the level of that electrode pair. Segmental volumes are calculated by multiplying the area by the electrode distance, with the total RV volume being the sum of these segments. After positioning, the catheter's placement is verified in the “segmental loops' view. Individual segmental loops should appear rectangular or trapezoidal and move counterclockwise on the conduct NT software. A clockwise rotation or “figure-of-8″ pattern suggests misplacement (e.g. location in the right atrium or at the level of the tricuspid valve or contact with RV wall, trabeculae, or moderator band). Malposition can appear in a single beat or across the entire recording. For proper analysis of RV function, at least three consecutive, valid segments are required (Fig. 1). Catheter position should be confirmed using angiography with contrast injection through the steerable guiding sheath. Invalid segments can be excluded for total volume at the catheter settings in the software, and adjustments can be made during post-processing. In case of insufficient number of segments, catheter repositioning may be necessary.

### 2.4 Measurements for volume calibration

Pressure calibration is automatically performed by the Conduct NT software when the pressure sensor is connected to the Inca system. In order to convert the measured conductance to volume, a calibration has to be performed. Volume calibration can be achieved by applying Fick's method to assess cardiac output (CO) and infusing hypertonic saline (10 ml 5 % NaCl) upstream from the conductance catheter to determine parallel conductance, providing calibration factors (SVcal and EFcal) to convert the conductance signal into absolute volumes. Hypertonic saline temporarily increases the sodium concentration in the blood, making it more conductive. Since hypertonic saline infusion does not significantly affect myocardial tissue conductivity (parallel conductance), accurate RV volume measurements can be obtained.

All measurements are performed pre and post-intervention during breath-hold to reduce respiratory variability and minimize the effects of intrathoracic pressure. Each measurement is repeated twice to ensure accuracy. Record for at least 20 s per measurement. Recordings should be free of extrasystoles or arrhythmias. If arrhythmias are detected during recording, measurements can either be repeated or corrected during post-processing, depending on the amount of artefacts. To obtain the calibration factor SVcal, perform a baseline “steady state' measurement. Start recording, induce a breath-hold, stop the breath-hold, stop recording, and repeat the process. Arterial and venous blood samples must be taken pre and post-intervention to measure oxygen saturations and hemoglobin levels, which are needed to calculate CO using Fick's method. If the catheter moves, a new baseline “steady state' measurement must be taken after repositioning. To obtain the calibration factor EFcal, perform a measurement with hypertonic saline (5 % NaCl) infusion. Start recording, induce a breath-hold, record at least 5 beats before injecting 10 ml of 5 % NaCl through the sheath side port in an accelerating fashion. Wait until the loop returns to baseline, then stop the breath-hold, stop recording, and repeat the process.

### 2.5 Measurements for preload alterations

Manipulation of the loading conditions of the heart are required to determine load-independent measures of RV function. There are three options to change preload: 1) fluid challenge, 2) balloon occlusion of the inferior vena cava, and 3) a passive leg raise maneuver. In our experience, the fluid challenge was ineffective, while balloon occlusion requires intervention. Given ethical concerns and variable success rates, we preferred passive leg raises as a non-invasive method to enhance preload [5]. Despite challenges with sterile fields and equipment, passive leg raise is effective to increase preload and can be safely performed, also in case of femoral vein access. Start recording, induce a breath-hold, record at least 5 beats before passively lifting both legs to a 45-degree angle, then continue recording and count approximately 15–20 beats from the moment leg lifting begins before lowering the legs. Wait for the loop to return to baseline, then stop the breath-hold, stop recording, and repeat the process. After measurements, record catheter removal to adjust for any atmospheric pressure offset during post-processing.

### 2.6 PV loop analysis

Prepare the files, exclude artifact beats or segments and complete volume calibration. Subsequently, select the beat from the last baseline to the maximum volume beat in the measurement with leg raises (ideally at least seven beats). This selection is used to assess intrinsic load-independent RV contractility (end-systolic elastance (Ees)), RV to pulmonary arterial (PA) coupling (Ees/Ea) and RV diastolic stiffness (end-diastolic elastance (Eed)) (Figure 1E-1 and 1E-2) . RV SV and CO are obtained from PV loop analysis, while RV end-diastolic volume, end-systolic volume and ejection fraction are recommended to be obtained from Cardiac Magnetic Resonance (CMR) imaging.

## 3 Clinical implications

Early identification of RV maladaptation is crucial since it is an independent predictor of prognosis [1]. RV PV loop analysis is considered the gold standard for evaluating RV adaptation to varying loading conditions, offering a load-independent assessment that accurately reflects intrinsic RV function. RV PV loop analysis using the multi-beat method can be safely and reliably applied in children and adults with complex CHD. Although currently used primarily for research purposes, the technique provides important insights into RV maladaptation in this population. This is especially important given the absence of non-invasive alternatives for RV PV loop analysis in CHD. Examining the effects of exercise and stress testing on PV loop measurements represents an important area for future research, as it may reveal RV dysfunction.

## 4 Experience with RV PV loops in CHD patients

Several studies have applied RV PV loop analysis using conductance catheters in patients with CHD, encompassing a broad spectrum of pathophysiologies such as single-ventricle physiology, a systemic RV and a subpulmonary morphological RV [[6], [7], [8], [9], [10]]. In a recent comprehensive review, Hiremath et al. summarized the clinical and research applications of PV loop analysis in CHD, highlighting how these measurements can provide condition-specific insights and guidance for interpretation across diverse physiologies [11].

## 5 Conclusion

RV PV loop analysis offers a valuable tool for advanced insight into RV adaptation and a deeper understanding of cardiac physiology in complex CHD patients with RV pressure and/or volume overload. By enhancing our understanding of RV adaptation, this approach holds great potential for improving patient management and optimizing treatment strategies.

## 7 Ethical approvement statement

This study trial was approved by the Medical Research Ethical Committee of the University Medical Center Utrecht, the Netherlands (METC NedMec).

## CRediT authorship contribution statement

**Renée S. Joosen:** Writing – review & editing, Writing – original draft, Visualization, Project administration, Methodology, Investigation, Formal analysis, Data curation, Conceptualization. **Michael G. Dickinson:** Writing – review & editing, Writing – original draft, Supervision, Software, Resources, Methodology, Investigation, Data curation, Conceptualization. **Marielle C. van de Veerdonk:** Writing – review & editing, Writing – original draft, Visualization, Supervision, Resources, Methodology, Investigation. **Rahi S. Alipour Symakani:** Writing – review & editing, Methodology, Investigation. **Daphne Merkus:** Writing – review & editing, Methodology, Investigation, Funding acquisition. **Michiel Voskuil:** Writing – review & editing, Writing – original draft, Supervision, Methodology, Investigation. **Gregor J. Krings:** Writing – review & editing, Writing – original draft, Supervision, Software, Resources, Methodology, Investigation. **Johannes M.P.J. Breur:** Writing – review & editing, Writing – original draft, Visualization, Supervision, Resources, Methodology, Investigation, Funding acquisition, Conceptualization.

## Patient consent statement

Informed consent was obtained from all patients.

## 6 Funding

We acknowledge the support from the Netherlands Cardiovascular Research Initiative to this article as funding source for R.S. Joosen and R.A. Symakani their PhD projects as part of the OUTREACH consortium: an initiative with support of the Dutch 10.13039/100002129Heart Foundation and Hartekind, CVON2019-002 OUTREACH. We also acknowledge the support from the German Center for Cardiovascular Research to D. Merkus (DZHK81Z0600207).

## Declaration of competing interest

The authors declare that they have no known competing financial interests or personal relationships that could have appeared to influence the work reported in this paper.
